# Supplementary material for: Socioeconomic and ethnical disparity in coronary heart disease outcomes in Denmark and the effect of cardiac rehabilitation—A nationwide registry study
Source: PLoS One. 2022 Nov 7;17(11):e0276768. doi: 10.1371/journal.pone.0276768 (PMC9639811; doi:10.1371/journal.pone.0276768)
Supplement: S1 File — (DOCX) [file pone.0276768.s001.docx]

# Supplementary tables and figures

## Table 1: Demographic characteristics of the DHRD population.

| Variables | Level | DHRD population |
| --- | --- | --- |
|  |  | N = 9,882 |
| Gender | Male | 7355 (74.4) |
| Age, mean (SD) |  | 64.9 (11.0) |
| Region | Capital | 2767 (28.0) |
|  | Zealand | 1817 (18.4) |
|  | Southern | 2569 (26.0) |
|  | Mid-Jutland | 2079 (21.0) |
|  | North-Jutland | 650 (6.6) |
| Diagnose | MI | 4567 (49.4) |
|  | Stable angina | 4674 (50.6) |
| Revascularized | Yes | 4290 (55.8) |
| Socioeconomic factors |  |  |
| Ethnicity | Denmark | 8928 (90.5) |
|  | Western | 328 (3.3) |
|  | Non-western | 614 (6.2) |
| Education | Basic | 3149 (31.9) |
|  | Short | 4474 (45.4) |
|  | Medium | 1655 (16.8) |
|  | High | 585 (5.9) |
| Living alone | Yes | 2964 (30.0) |
| Work status | Self-employed | 381 (3.9) |
|  | Employee | 3140 (31.8) |
|  | Unemployed/early retirement | 1084 (11.0) |
|  | Retiree | 5154 (52.2) |
| Income (quartiles) | 0-25 | 2134 (21.6) |
|  | 25-50 | 2092 (21.2) |
|  | 50-75 | 2435 (24.6) |
|  | 75-100 | 3220 (32.6) |
| Comorbidity |  |  |
| Known hypertension |  | 7179 (72.6) |
| Known hypercholesterolemia |  | 6029 (61.0) |
| Known diabetes |  | 1563 (15.8) |
| Known CHD |  | 2626 (28.1) |
| Charlson comorbidity index | Score >3 | 665 (6.7) |

Data are presented as n (%) unless otherwise specified. Abbreviations: SD: Standard deviation, MI: acute myocardial infarction, CHD: coronary heart disease, COPD: chronic obstructive pulmonary disease, wCI: wheighted Charlson comorbidity index

## Table 2: Quality indicators in the DHRD population

## By education

| Variable | Basic  education | Short education | Medium education | High education | p-value |
| --- | --- | --- | --- | --- | --- |
|  | 3149 | 4474 | 1655 | 585 |  |
| Quality indicators |  |  |  |  |  |
| Participated in >80% of exercise sessions | 586 (67.8) | 992 (70.6) | 382 (70.2) | 101 (62.0) | 0.534* |
| VO_2peak_ increased >10% | 144 (42.7) | 271 (46.4) | 123 (49.2) | 48 (53.9) | **0.006*** |
| Smoking cessation among smokers at baseline | 363 (38.5) | 513 (42.5) | 161 (45.9) | 42 (34.7) | **<0.001*** |
| Received dietary consulting | 231 (22.1) | 317 (21.8) | 124 (24.6) | 23 (17.6) | 0.155* |
| Achieved LDL goal | 1683 (65.4) | 2455 (68.2) | 875 (68.8) | 262 (72.2) | 0.110* |
| Achieved BP goal | 1841 (70.6) | 2562 (70.2) | 915 (70.9) | 270 (72.8) | 0.605* |
| Screened for diabetes | 1833 (77.2) | 2621 (76.4) | 981 (75.3) | 262 (57.0) | 0.325* |
| Screened for depression | 1605 (61.7) | 2282 (62.7) | 828 (64.4) | 259 (70.4) | 0.336* |
| ASA or AC at end of CR | 2866 (91.0) | 4107 (91.8) | 1539 (93.0) | 536 (91.6) | 0.073* |
| Statin at end of CR | 2503 (96.1) | 3504 (96.1) | 1228 (95.5) | 356 (96.0) | 0.636* |
| Betablockers at end of CR | 1805 (69.3) | 2462 (67.5) | 858 (66.7) | 237 (63.9) | 0.103* |

## By ethnicity

| Variable | Denmark | Western countries | Non-western countries | p-value |
| --- | --- | --- | --- | --- |
|  | 8928 | 328 | 614 |  |
| Quality indicators |  |  |  |  |
| Participated in >80% of exercise sessions | 1905 (69.4) | 59 (67.8) | 96 (64.9) | 0.712* |
| VO_2peak_ increased >10% | 544 (46.6) | 16 (53.3) | 25 (41.0) | 0.794* |
| Smoking cessation among smokers at baseline | 969 (42.4) | 44 (40.4) | 70 (30.3) | 0.107* |
| Received dietary consulting | 656 (22.2) | 25 (29.4) | 13 (12.6) | **0.002*** |
| Achieved LDL goal | 4851 (67.3) | 143 (64.1) | 290 (75.1) | 0.196* |
| Achieved BP goal | 5123 (70.1) | 159 (69.4) | 312 (79.6) | 0.098* |
| Screened for diabetes | 5300 (76.5) | 178 (67.2) | 227 (59.9) | 0.079* |
| Screened for depression | 4595 (63.0) | 147 (64.8) | 237 (60.6) | **<0.001*** |
| ASA or AC at end of CR | 8213 (91.7) | 309 (94.1) | 553 (90.3) | 0.162* |
| Statin at end of CR | 7010 (96.0) | 213 (93.4) | 379 (96.9) | 0.499* |
| Betablockers at end of CR | 4937 (67.6) | 157 (68.9) | 273 (69.8) | 0.157* |

*p-value adjusted for gender, age and center. Data are presented as n (%) unless otherwise specified. Abbreviations: VO_2peak_: peak oxygen consumption, LDL: Low density lipoprotein, BP: blood pressure, ASA; acetylsalicylic acid, AC: anticoagulants, CR: cardiac rehabilitation

## Table 3: Demographics of the CR participants defined by the DNPR. Stratified on education and ethnicity.

| N total: 19,383 | By | education |  |  | By | ethnicity |  |
| --- | --- | --- | --- | --- | --- | --- | --- |
|  | Basic | Short | Medium | High | Danish | Western | Non-western |
| Variable | 6,417 | 7,569 | 2,544 | 806 | 16,278 | 496 | 913 |
| Age, median (IQR) | 69.5 (12.5) | 66.1 (11.4) | 66.6 (11.3) | 66.3 (10.5) | 67.8 (11.9) | 64.7 (12.6) | 59.7 (11.1) |
| Gender male | 4102 (63.9) | 5942 (78.5) | 1805 (71.0) | 700 (86.8) | 11684 (71.8) | 360 (72.6) | 753 (82.5) |
| MI | 3713 (57.9) | 4244 (56.1) | 1404 (55.2) | 413 (51.2) | 9247 (56.8) | 295 (59.5) | 455 (49.8) |
| PCI | 2876 (44.8) | 3376 (44.6) | 1119 (44.0) | 301 (37.5) | 7280 (44.7) | 178 (35.9) | 343 (37.6) |
| CABG | 903 (14.1) | 1335 (17.6) | 468 (18.4) | 208 (25.8) | 2656 (16.3) | 92 (18.5) | 225 (24.6) |
| CVD risk factors |  |  |  |  |  |  |  |
| Hypertension | 5015 (78.2) | 5656 (74.7) | 1828 (71.9) | 546 (67.7) | 12283 (75.5) | 356 (71.8) | 664 (72.7) |
| Hypercholestrolemia | 4217 (65.7) | 4827 (63.8) | 1595 (62.7) | 483 (59.9) | 10398 (63.9) | 292 (58.9) | 645 (70.6) |
| Diabetes | 1455 (22.7) | 1416 (18.7) | 373 (14.7) | 126 (15.6) | 3036 (18.7) | 74 (14.9) | 345 (37.8) |
| Known CHD | 2784 (43.4) | 2990 (39.5) | 996 (39.2) | 291 (36.1) | 6601 (40.6) | 202 (40.7) | 416 (45.6) |
| Comorbidity |  |  |  |  |  |  |  |
| Charlson wCI, mean (SD) | 1.97 (1.74) | 1.73 (1.60) | 1.58 (1.52) | 1.58 (1.60) | - 1. 1.66) | 1.64 (1.52) | 1.63 (1.49) |

Data are presented as n (%) unless otherwise specified. Abbreviations: IQR; Interquartile range, SD: Standard deviation, MI: acute myocardial infarction, PCI: percutaneous coronary intervention, CABG: coronary artery bypass graft, CVD: cardiovascular disease, CHD: coronary heart disease, wCI: wheighted Charlson comorbidity index

## Table 4. Hazard ratios (95% Confidence Intervals) from Cox-regression models for the combined endpoint (MACE and all-cause mortality) by education and ethnicity. Stratified on CR participation.

| *CR participants reference (base)* | Crude HR (95% CI) | p-value | Adjusted HR (95% CI)* | p-value |
| --- | --- | --- | --- | --- |
| By education |  |  |  |  |
| Basic education | 1.0 (0.95-1.11) | 0.456 | 1.0 (0.94-1.1) | 0.606 |
| Short education | 1.1 (1.0-1.2) | 0.060 | 1.0 (0.94-1.1) | 0.509 |
| Medium education | 1.0 (0.87-1.2) | 0.903 | 0.96 (0.83-1.1) | 0.622 |
| High education | 1.1 (0.85-1.4) | 0.463 | 1.2 (0.89-1.5) | 0.276 |
| By ethnicity |  |  |  |  |
| Danish origin | 1.1 (1.0-1.1) | **0.018** | 1.0 (0.96-1.1) | 0.458 |
| Western origin | 1.1 (0.80-1.4) | 0.600 | 1.0 (0.77-1.4) | 0.856 |
| Non-western origin | 0.98 (0.80-1.2) | 0.894 | 0.99 (0.81-1.2) | 0.963 |

*adjusted for age, gender, comorbidity index, hypertension, diabetes, hypercholesterolemia and index-event. Abbreviations: CR; Cardiac Rehabilitation, HR; Hazard Ration, CI; Confidence interval

#

## Table 5. Hazard ratios (95% Confidence Intervals) from Cox-regression models for the combined endpoint (MACE and all-cause mortality) by age-groups in CR participants stratified on education and ethnicity.

|  | Crude HR | (95% CI) |  | Adjusted HR | (95% CI)* |  |
| --- | --- | --- | --- | --- | --- | --- |
|  | Age <60 | Age 60-79 | Age >79 | Age <60 | Age 60-79 | Age >79 |
| By education | N=4,357 | N=10,751 | N=4,257 | N=4,357 | N=10,751 | N=4,257 |
| Basic education | 1 (base) |  |  |  |  |  |
| Short education | **.75 (.63-.90)^b^** | **.81 (.73-.89)^a^** | .94 (.81-1.1) | **.78 (.66-.95)^a^** | **.84 (.76-.92)^a^** | .99 (.85-1.1) |
| Medium education | .87 (.68-1.1) | **.75 (.65-.86) ^a^** | **.76 (.59-.97)^a^** | .95 (.74-1.2) | **.82 (.71-.94)^a^** | .82 (.64-1.0) |
| High education | **.48 (.28-.81)^b^** | **.70 (.55-a.88) ^a^** | .79 (.50-1.2 | .54 (.32-.91)^a^ | **.71 (.56-.90)^a^** | .86 (.55-1.4) |
| By ethnicity |  |  |  |  |  |  |
| Danish origin | 1 (base) |  |  |  |  |  |
| Western origin | .87 (.54-.1.4) | 1.19 (.84-1.4) | .97 (.58-1.6) | .96 (.60-1.5) | 1.16 (.89-1.5) | .83 (.49-1.4) |
| Non-western origin | **1.3 (1.1-1.7)^a^** | 1.15 (.93-1.4) | 1.2 (.69-2.2) | **1.3 (1.1-1.6)^b^** | 1.20 (.97-1.5) | 1.4 (.77-2.4) |

^a^=P<0.05, ^b^=P<0.01 ^c^=P<0.001

Abbreviations: HR; Hazard Ration, CI; Confidence interval

## Table 6. Hazard ratios (95% Confidence Intervals) from Cox-regression models for the combined endpoint (MACE and all-cause mortality) by age-groups in CR participants stratified on index event.

|  | Crude HR | (95% CI) |  | Adjusted HR | (95% CI)* |  |
| --- | --- | --- | --- | --- | --- | --- |
|  | MI | CABG | PCI | MI | CABG | PCI |
| By education | N=10,123 | N=3,583 | N=8,824 | N=10,123 | N=3,583 | N=8,824 |
| Basic education | 1 (base) |  |  |  |  |  |
| Short education | **.74 (.69-.81)^c^** | **.80 (.65-.99)^b^** | **.82 (.73-.92)^b^** | **.86 (.79-.93) ^c^** | .83(.68-1.0) | **.86 (.77-.97)^b^** |
| Medium education | **.66 (.58-.73) ^c^** | .88 (.67-1.2) | **.81 (.69-.96)^b^** | **.78 (.70-.88) ^c^** | .93(.71-1.2) | .89 (.76-1.0) |
| High education | **.62 (.50-.75)^c^** | .81 (.55-1.2) | .75 (.55-1.0) | **.74 (.60-.91)^b^** | .86 (.58-1.3) | .76 (.57-1.0) |
| By ethnicity |  |  |  |  |  |  |
| Danish origin | 1 (base) |  |  |  |  |  |
| Western origin | .89 (.72-.1.1) | .78 (.44-1.4) | 1.1 (.78-1.5) | 1.0 (.84-1.3) | .86 (.48-1.5) | 1.1 (.82-1.6) |
| Non-western origin | **.81 (.68-.96)^b^** | 1.0 (.72-1.4) | 1.2 (.97-1.5) | 1.2 (1.0-1.4) | .99 (.70-1.4) | **1.4 (1.1-1.8)^b^** |

^a^=P<0.05, ^b^=P<0.01 ^c^=P<0.001

Abbreviations: HR; Hazard Ration, CI; Confidence interval, MI: acute myocardial infarction, PCI: percutaneous coronary intervention, CABG: coronary artery bypass graft

## Table 7: Hazard ratios (95% Confidence Intervals) from Cox-regression models for the combined endpoint (MACE and all-cause mortality) by age-groups across education and ethnicity. Stratified on CR participation.

| *CR participants reference (base)* | Crude HR | (95% CI) |  | Adjusted HR | (95% CI)* |  |
| --- | --- | --- | --- | --- | --- | --- |
| By education | Age <59  N= 8,056 | Age 60-79  N= 18,282 | Age >79  N= 8,173 | Age <59  N= 8,056 | Age 60-79  N= 18,282 | Age >79  N= 8,173 |
| Basic education | .96 (.79-1.2) | 1.0 (.93-1.7) | 1.0 (.88-1.2) | .94 (.78-1.2) | 1.0 (-91-1.1) | 1.0 (.89-1.2) |
| Short education | 1.1 (.91-1.3) | 1.06 (.96-1.2) | .97 (.81-1.2) | 1.1 (.88-1.3) | 1.0 (.92-1.1) | .92 (.78-1.1) |
| Medium education | .87 (.63-1.2) | 1.02 (.84-1.23) | 1.1 (.79-1.5) | .82 (.60-1.1) | .98 (.80-1.2) | .96 (.69-1.3) |
| High education | 1.2 (.62-2.4) | 1.04 (.75-1.45) | 1.4 (.79-2.6) | 1.2 (.60-2.3) | 1.1 (.80-1.6) | 1.0 (.55-1.9) |
| By ethnicity |  |  |  |  |  |  |
| Danish origin | 1.0 (.88-1.2) | 1.05 (.98-1.1) | 1.0 (.92-1.12) | .97 (.85-1.1) | 1.0 (.95-1.1) | .98 (.89-1.1) |
| Western origin | .79 (.39-1.6) | 1.06 (.73-1.5) | 1.4 (.74-1.23) | .69 (.33-1.42) | 1.0 (.72-1.5) | 1.4 (.72-2.7) |
| Non-western origin | 1.0 (.77-1.42) | 1.0 (.75-1.4) | .52 (.21-1.23) | 1.1 (.78-1.4) | 1.0 (.76-1.4) | .47 (.19-1.2) |

^a^=P<0.05, ^b^=P<0.01 ^c^=P<0.001

Abbreviations: CR; Cardiac Rehabilitation, HR; Hazard Ration, CI; Confidence interval

## Table 8: Hazard ratios (95% Confidence Intervals) from Cox-regression models for the combined endpoint (MACE and all-cause mortality) by index event across education and ethnicity. Stratified on CR participation.

| *CR participants reference (base)* | Crude HR | (95% CI) |  | Adjusted HR | (95% CI)* |  |
| --- | --- | --- | --- | --- | --- | --- |
|  | AMI | CABG** | PCI | AMI | CABG** | PCI |
| By education | N=19,248 | N=3,698 | N=15,778 | N=19,248 | N=3,698 | N=15,778 |
| Basic education | .92 (.83-1.0) |  | .97 (.85-1.1) | .96 (.86-1.1) |  | .97 (.85-1.1) |
| Short education | **.88 (.79-.99)^a^** |  | .99 (.88-1.1) | **.83 (.75-.93)^b^** |  | .97 (.86-1.1) |
| Medium education | .95 (.77-1.2) |  | .93 (.75-1.2) | .92 (.74-1.1) |  | .90 (.72-1.1) |
| High education | 1.2 (.80-1.7) |  | .85 (.57-1.2) | 1.2 (.79-1.7) |  | .93 (.62-1.4) |
| By ethnicity |  |  |  |  |  |  |
| Danish origin | .93 (.86-1.0) |  | .98 (.90-1.1) | **.91 (.84-.97)^a^** |  | .97 (.89-1.1) |
| Western origin | .87 (.58-.1.3) |  | .87 (.54-1.4) | .87 (.57-1.3) |  | .89 (.56-1.4) |
| Non-western origin | .98 (.71-1.4) |  | .80 (.59-1.1) | .99 (.71-1.4) |  | .81 (.60-1.1) |

**groups too small for analysis ^a^=P<0.05, ^b^=P<0.01 ^c^=P<0.001

Abbreviations: HR; Hazard Ration, CI; Confidence interval, MI: acute myocardial infarction, PCI: percutaneous coronary intervention, CABG: coronary artery bypass graft

## Figure 1

## Flowchart of population defined by DNPR; patients with MI/revascularization

Abbreviations: DNPR; Danish national patients registry, CABG; coronary artery bypass, PCI; percutaneous coronary intervention

## Figure 2

## Diagram of the overlap between the DNPR and DHRD population

Abbreviations: N; number, MI; myocardial infarction, CABG; coronary artery bypass, PCI; percutaneous coronary intervention, DNPR; Danish national patient register, CR; cardiac rehabilitation, DHRD; Danish cardiac rehabilitation database
